# The genome sequence of the furrow orbweaver, *Larinioides cornutus *(Clerck, 1757)

**DOI:** 10.12688/wellcomeopenres.24288.1

**Published:** 2025-05-23

**Authors:** Mark G. Telfer, Liam M. Crowley, Craig S. Wilding, University of Oxford and Wytham Woods Genome Acquisition Lab, Darwin Tree of Life Barcoding collective, Wellcome Sanger Institute Tree of Life Management, Samples and Laboratory team, Wellcome Sanger Institute Scientific Operations: Sequencing Operations, Wellcome Sanger Institute Tree of Life Core Informatics team, Tree of Life Core Informatics collective, Darwin Tree of Life Consortium

**Affiliations:** 1 Independent researcher, Ventnor, Isle of Wight, England, UK; 2 Department of Biology, University of Oxford, Oxford, England, UK; 3 Schoold of Biology and Environmental Science, University College Dublin, Dublin, Leinster, Ireland

**Keywords:** Larinioides cornutus, furrow orbweaver, genome sequence, chromosomal, Araneae

## Abstract

We present a genome assembly from a female specimen of *Larinioides cornutus* (furrow orbweaver; Arthropoda; Arachnida; Araneae; Araneidae). The assembly contains two haplotypes with total lengths of 2,304.58 megabases and 2,295.32 megabases. Most of haplotype 1 (98.49%) is scaffolded into 13 chromosomal pseudomolecules, including the X _1_ and X _2_ sex chromosomes. Haplotype 2 was assembled to scaffold level. The mitochondrial genome has also been assembled, with a length of 14.6 kilobases.

## Species taxonomy

Eukaryota; Opisthokonta; Metazoa; Eumetazoa; Bilateria; Protostomia; Ecdysozoa; Panarthropoda; Arthropoda; Chelicerata; Arachnida; Araneae; Araneomorphae; Entelegynae; Orbiculariae; Araneoidea; Araneidae; *Larinioides*; *Larinioides cornutus* (Clerck, 1757) (NCBI:txid336591)

## Background

Of the three members of the genus *Larinioides* found in the UK and Ireland ( Bee *et al.*, 2020; Lavery, 2019), *Larinioides cornutus* is both the most common and most widespread. It has a holoarctic distribution, being found from North America, through Europe, Russia, areas of the Middle East and into Asia ( https://wsc.nmbe.ch/species/4277/Larinioides_cornutus). In the UK, it can be found throughout England and Wales but has a more scattered distribution in Scotland. On the island of Ireland, records exist for all but four Irish, and two Northern Irish counties ( Van Helsdingen, 1996), although these gaps are likely reflective of gaps in sampling effort or reporting.

*Larinioides cornutus* reaches sizes of 5–8 mm (♂) and 6–9 mm (♀) ( Bee *et al.*, 2020; Locket & Millidge, 1951; Roberts, 1987), and has a pale brown carapace covered with white hairs, and pale, sometimes annulated legs. The abdomen is variable in colour; from cream through to orange-brown and is decorated with an obvious dark folium with light transverse stripes posteriorly, and a pale cardiac region with central dark patch ( Bee *et al.*, 2020; Locket & Millidge, 1951; Roberts, 1987).

This species is typically found close to water on tall grasses and reeds, as well as bridges and posts, where it spins large, sometimes prominent webs; however, it may be found also in damp, rough grassland and meadows ( Bee *et al.*, 2020; Harvey *et al.*, 2002). The web may appear empty during the day since the spider hides in a silken retreat constructed high up in vegetation adjacent to the web and camouflaged with plant and animal matter, emerging only at night.

**Figure 1.**
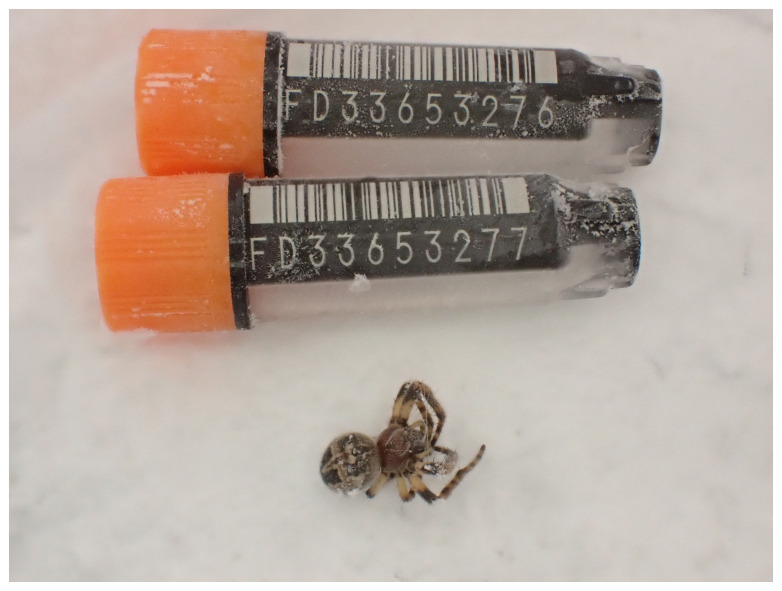
Photograph of the *Larinioides cornutus* (qqLarCorn3) specimen used for genome sequencing.

Whilst a transcriptome for *L. cornutus* has been produced previously ( Wilson *et al.*, 2020), no genome resource exists. To this end, we present a chromosomally complete genome sequence for *L. cornutus* based on an individual ( Figure 1) collected from Cothill Fen, Oxfordshire. This will aid in future population genetic studies of this widespread species, as well as contributing to broader studies of the biology and phylogeny of the order Araneae ( Garb *et al.*, 2018).

## Genome sequence report

### Sequencing data

The genome of a specimen of *Larinioides cornutus* ( Figure 1) was sequenced using Pacific Biosciences single-molecule HiFi long reads, generating 67.91 Gb from 8.03 million reads, which were used to assemble the genome. GenomeScope analysis estimated the haploid genome size at 2,258.74 Mb, with a heterozygosity of 2.43% and repeat content of 27.74%. These estimates guided expectations for the assembly. Based on the estimated genome size, the sequencing data provided approximately 29x coverage. Hi-C sequencing produced 480.07 Gb from 3,179.24 million reads and was used to scaffold the assembly. Table 1 summarises the specimen and sequencing details.

**Table 1.** Specimen and sequencing data for *Larinioides cornutus*.

Project information			
**Study title**	Larinioides cornutus		
**Umbrella BioProject**	PRJEB82167		
**Species**	*Larinioides cornutus*		
**BioSpecimen**	SAMEA114644584		
**NCBI taxonomy ID**	336591		
**Specimen information**			
**Technology**	**ToLID**	**BioSample accession**	**Organism part**
**PacBio long read sequencing**	qqLarCorn3	SAMEA114645200	head and thorax
**Hi-C sequencing**	qqLarCorn3	SAMEA114645200	head and thorax
**Sequencing information**			
**Platform**	**Run accession**	**Read count**	**Base count (Gb)**
**Hi-C Illumina NovaSeq 6000**	ERR13947480	1.58e+09	239.33
**Hi-C Illumina NovaSeq X**	ERR13947481	1.59e+09	240.74
**PacBio Revio**	ERR13946461	8.03e+06	67.91

### Assembly statistics

The genome was assembled into two haplotypes using Hi-C phasing. Haplotype 1 was curated to chromosome level, while haplotype 2 was assembled to scaffold level. The assembly was improved by manual curation, which corrected 96 misjoins or missing joins. These interventions decreased the scaffold count by 6.52%. The final assembly has a total length of 2,304.58 Mb in 501 scaffolds, with 732 gaps, and a scaffold N50 of 175.46 Mb ( Table 2).

**Table 2.** Genome assembly data for *Larinioides cornutus*.

Genome assembly	Haplotype 1	Haplotype 2
Assembly name	qqLarCorn3.hap1.1	qqLarCorn3.hap2.1
Assembly accession	GCA_964340895.1	GCA_964340905.1
Assembly level	chromosome	scaffold
Span (Mb)	2,304.58	2,295.32
Number of contigs	1,233	1,568
Number of scaffolds	501	871
Longest scaffold (Mb)	195.78	-
Assembly metrics (benchmark)	Haplotype 1	Haplotype 2
Contig N50 length (≥ 1 Mb)	5.57 Mb	5.23 Mb
Scaffold N50 length (= chromosome N50)	175.46 Mb	171.8 Mb
Consensus quality (QV) (≥ 40)	61.9	61.7
*k*-mer completeness	64.94%	64.69%
Combined *k*-mer completeness (≥ 95%)	99.59%	
BUSCO * (S > 90%; D < 5%)	C:98.2%[S:92.5%,D:5.6%], F:0.8%,M:1.1%,n:2,934	C:98.1%[S:92.4%,D:5.7%], F:0.7%,M:1.2%,n:2,934
Percentage of assembly mapped to chromosomes (≥ 90%)	98.49%	-
Sex chromosomes (localised homologous pairs)	X _1_ and X _2_	-
Organelles (one complete allele)	Mitochondrial genome: 14.6 kb	-

*BUSCO scores based on the arachnida_odb10 BUSCO set using version 5.5.0. C = complete [S = single copy, D = duplicated], F = fragmented, M = missing, n = number of orthologues in comparison.

**Figure 2.**
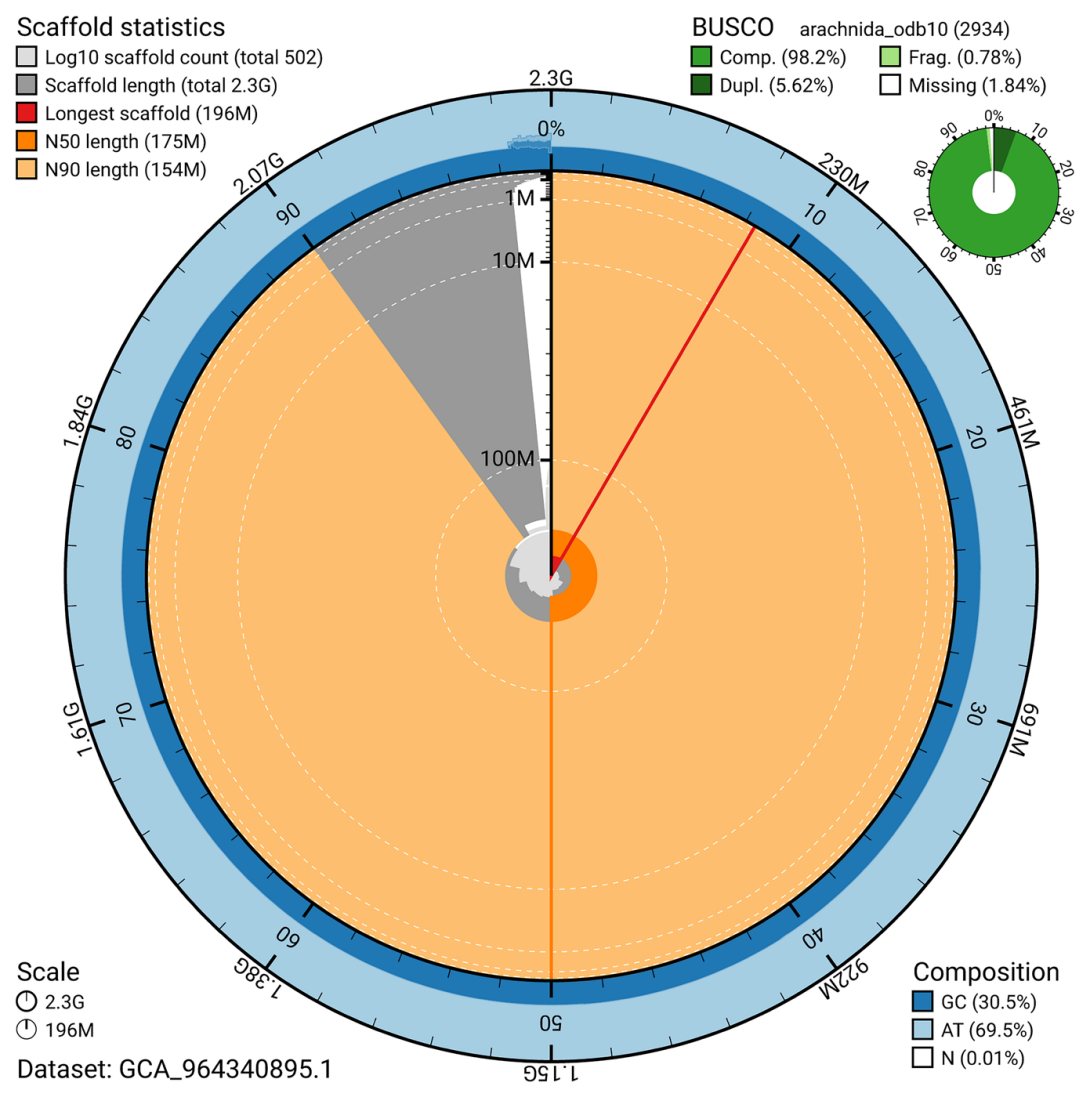
Genome assembly of *Larinioides cornutus*, qqLarCorn3.hap1.1: metrics. The BlobToolKit snail plot provides an overview of assembly metrics and BUSCO gene completeness. The circumference represents the length of the whole genome sequence, and the main plot is divided into 1,000 bins around the circumference. The outermost blue tracks display the distribution of GC, AT, and N percentages across the bins. Scaffolds are arranged clockwise from longest to shortest and are depicted in dark grey. The longest scaffold is indicated by the red arc, and the deeper orange and pale orange arcs represent the N50 and N90 lengths. A light grey spiral at the centre shows the cumulative scaffold count on a logarithmic scale. A summary of complete, fragmented, duplicated, and missing BUSCO genes in the arachnida_odb10 set is presented at the top right. An interactive version of this figure is available at https://blobtoolkit.genomehubs.org/view/GCA_964340895.1/dataset/GCA_964340895.1/snail.

**Figure 3.**
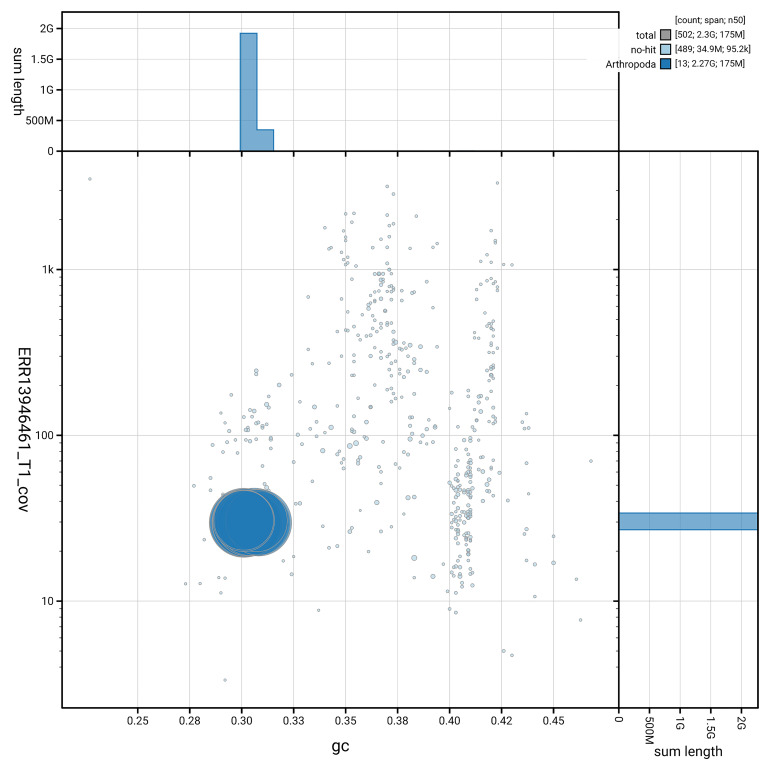
Genome assembly of *Larinioides cornutus*, qqLarCorn3.hap1.1: BlobToolKit GC-coverage plot. Blob plot showing sequence coverage (vertical axis) and GC content (horizontal axis). The circles represent scaffolds, with the size proportional to scaffold length and the colour representing phylum membership. The histograms along the axes display the total length of sequences distributed across different levels of coverage and GC content. An interactive version of this figure is available at https://blobtoolkit.genomehubs.org/view/GCA_964340895.1/dataset/GCA_964340895.1/blob.

**Figure 4.**
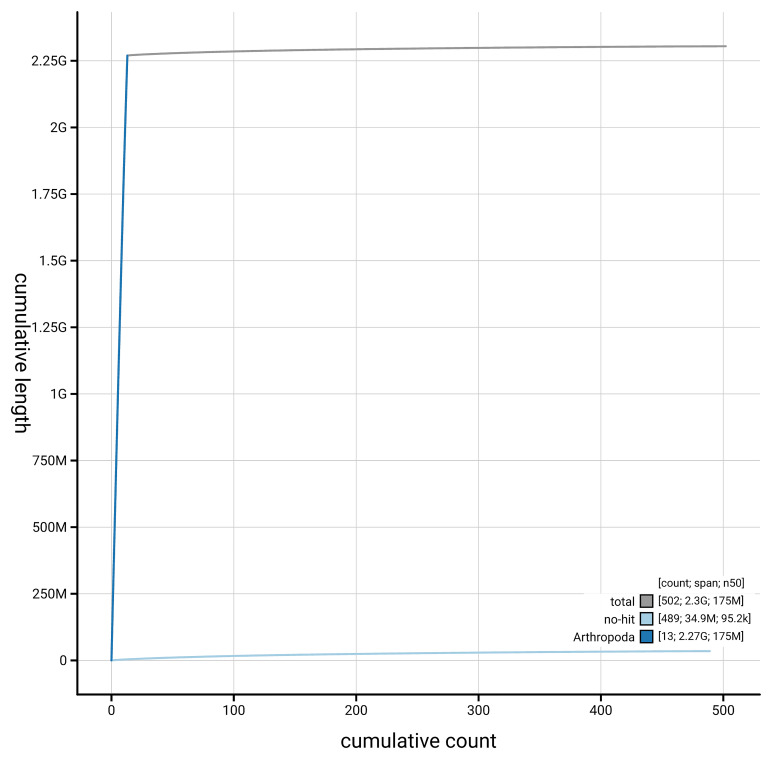
Genome assembly of *Larinioides cornutus,* qqLarCorn3.hap1.1: BlobToolKit cumulative sequence plot. The grey line shows cumulative length for all scaffolds. Coloured lines show cumulative lengths of scaffolds assigned to each phylum using the buscogenes taxrule. An interactive version of this figure is available at https://blobtoolkit.genomehubs.org/view/GCA_964340895.1/dataset/GCA_964340895.1/cumulative.

The snail plot in Figure 2 provides a summary of the assembly statistics, indicating the distribution of scaffold lengths and other assembly metrics. Figure 3 shows the distribution of scaffolds by GC proportion and coverage. Figure 4 presents a cumulative assembly plot, with separate curves representing different scaffold subsets assigned to various phyla, illustrating the completeness of the assembly.

**Figure 5.**
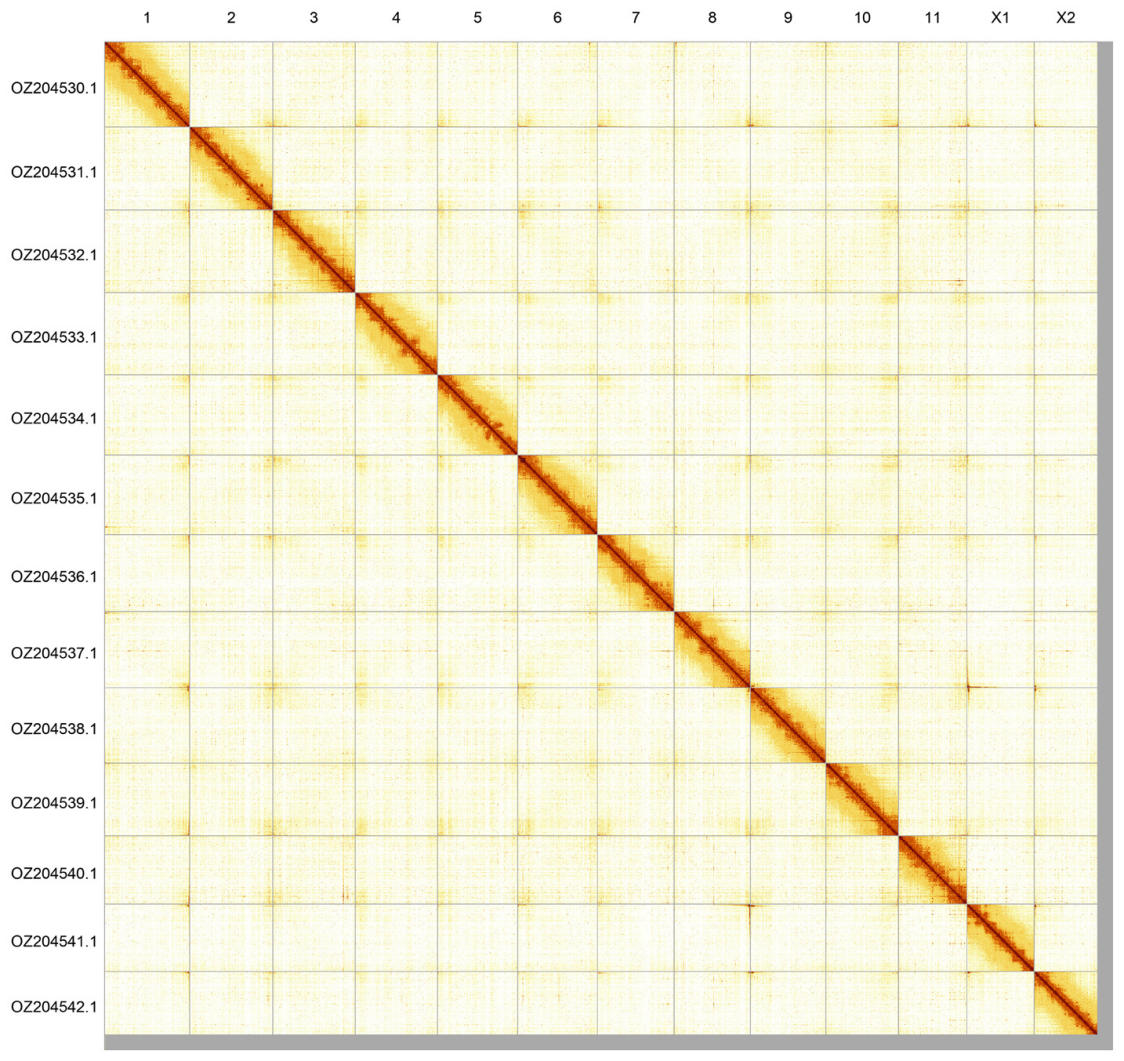
Genome assembly of *Larinioides cornutus*. Hi-C contact map of the qqLarCorn3.hap1.1 assembly, generated using PretextSnapshot. Chromosomes are shown in order of size and labelled with chromosome numbers (top) and chromosome accession numbers (left).

Most of the assembly sequence (98.49%) was assigned to 13 chromosomal-level scaffolds, representing 11 autosomes and the X _1_ and X _2_ sex chromosomes. These chromosome-level scaffolds, confirmed by Hi-C data, are named according to size ( Figure 5; Table 3). During curation, the X _1_ and X _2_ chromosomes were assigned based on homology to the genome of *Gibbaranea gibbosa* (GCA_964059485.1) ( Crowley *et al.*, 2025).

**Table 3.** Chromosomal pseudomolecules in the genome assembly of *Larinioides cornutus*, qqLarCorn3.

INSDC accession	Name	Length (Mb)	GC%
OZ204530.1	1	195.78	30
OZ204531.1	2	189.23	30
OZ204532.1	3	188.7	30.5
OZ204533.1	4	188.04	30
OZ204534.1	5	183.06	30
OZ204535.1	6	182.09	31
OZ204536.1	7	175.46	30.5
OZ204537.1	8	174.12	30.5
OZ204538.1	9	172.09	30.5
OZ204539.1	10	165.83	31
OZ204540.1	11	156.76	30.5
OZ204543.1	MT	0.01	22.5
OZ204541.1	X _1_	153.77	30
OZ204542.1	X _2_	144.78	30

The mitochondrial genome was also assembled. This sequence is included as a contig in the multifasta file of the genome submission and as a standalone record (Accession number OZ204543.1).

### Assembly quality metrics

The estimated Quality Value (QV) and *k*-mer completeness metrics, along with BUSCO completeness scores, were calculated for each haplotype and the combined assembly. The QV reflects the base-level accuracy of the assembly, while *k*-mer completeness indicates the proportion of expected *k*-mers identified in the assembly. BUSCO scores provide a measure of completeness based on benchmarking universal single-copy orthologues.

For haplotype 1, the estimated QV is 61.9, and for haplotype 2, 61.7. When the two haplotypes are combined, the assembly achieves an estimated QV of 61.8. The *k*-mer recovery for haplotype 1 is 64.94%, and for haplotype 2 64.69%, while the combined haplotypes have a *k*-mer recovery of 99.59%. BUSCO v.5.5.0 analysis using the arachnida_odb10 reference set ( *n* = 2,934) identified 98.2% of the expected gene set (single = 92.5%, duplicated = 5.6%) for haplotype 1.

Table 2 provides assembly metric benchmarks adapted from Rhie *et al.* (2021) and the Earth BioGenome Project Report on Assembly Standards September 2024. The haplotype 1 assembly achieves the EBP reference standard of **6.C.Q61**.

## Methods

### Sample acquisition and DNA barcoding

The specimen used for genome sequencing was an adult female *Larinioides cornutus* (specimen ID Ox003518, ToLID qqLarCorn3), collected from Cothill Fen, Oxforshire, United Kingdom (latitude 51.695, longitude –1.335) on 2023-05-12, using a sweep net. The specimen was collected by Mark Telfer and Liam Crowley (University of Oxford) and identified by Liam Crowley, and then preserved on dry ice.

The initial identification was verified by an additional DNA barcoding process according to the framework developed by Twyford *et al.* (2024). A small sample was dissected from the specimen and stored in ethanol, while the remaining parts were shipped on dry ice to the Wellcome Sanger Institute (WSI) ( Pereira *et al.*, 2022). The tissue was lysed, the COI marker region was amplified by PCR, and amplicons were sequenced and compared to the BOLD database, confirming the species identification ( Crowley *et al.*, 2023). Following whole genome sequence generation, the relevant DNA barcode region was also used alongside the initial barcoding data for sample tracking at the WSI ( Twyford *et al.*, 2024). The standard operating procedures for Darwin Tree of Life barcoding have been deposited on protocols.io ( Beasley *et al.*, 2023).

Metadata collection for samples adhered to the Darwin Tree of Life project standards described by Lawniczak *et al*. (2022).

### Nucleic acid extraction

The workflow for high molecular weight (HMW) DNA extraction at the Wellcome Sanger Institute (WSI) Tree of Life Core Laboratory includes a sequence of procedures: sample preparation and homogenisation, DNA extraction, fragmentation and purification ( Howard *et al.*, 2025). Detailed protocols are available on protocols.io ( Denton *et al.*, 2023b). The qqLarCorn3 sample was prepared for DNA extraction by weighing and dissecting it on dry ice ( Jay *et al.*, 2023). Tissue from the cephalothorax was homogenised using a PowerMasher II tissue disruptor ( Denton *et al.*, 2023a).

HMW DNA was extracted in the WSI Scientific Operations core using the Automated MagAttract v2 protocol ( Oatley *et al.*, 2023). The DNA was sheared into an average fragment size of 12–20 kb in a Megaruptor 3 system ( Bates *et al.*, 2023). Sheared DNA was purified by solid-phase reversible immobilisation, using AMPure PB beads to eliminate shorter fragments and concentrate the DNA ( Strickland *et al.*, 2023). The concentration of the sheared and purified DNA was assessed using a Nanodrop spectrophotometer and Qubit Fluorometer using the Qubit dsDNA High Sensitivity Assay kit. Fragment size distribution was evaluated by running the sample on the FemtoPulse system.

### Hi-C sample preparation and crosslinking

Hi-C data were generated from the qqLarCorn3 sample using the Arima-HiC v2 kit (Arima Genomics) with 20–50 mg of frozen tissue (stored at –80 °C). As per manufacturer’s instructions, tissue was fixed, and the DNA crosslinked using a TC buffer with a final formaldehyde concentration of 2%. The tissue was then homogenised using the Diagnocine Power Masher-II. The crosslinked DNA was digested using a restriction enzyme master mix, then biotinylated and ligated. A clean up was performed with SPRIselect beads prior to library preparation. DNA concentration was quantified using the Qubit Fluorometer v4.0 (Thermo Fisher Scientific) and Qubit HS Assay Kit, and sample biotinylation percentage was estimated using the Arima-HiC v2 QC beads.

### Library preparation and sequencing

Library preparation and sequencing were performed at the WSI Scientific Operations core.

***PacBio HiFi***

Samples with an average fragment size greater than 8 kb and total mass exceeding 400 ng were eligible for the low-input SMRTbell Prep Kit 3.0 protocol (Pacific Biosciences, California, USA), depending on genome size and required sequencing depth. Libraries were prepared using the SMRTbell Prep Kit 3.0 according to the manufacturer’s instructions. The kit includes reagents for end repair/A-tailing, adapter ligation, post-ligation SMRTbell bead clean-up, and nuclease treatment. Size selection and clean-up were performed using diluted AMPure PB beads (Pacific Biosciences). DNA concentration was quantified using a Qubit Fluorometer v4.0 (ThermoFisher Scientific) and the Qubit 1X dsDNA HS assay kit. Final library fragment size was assessed with the Agilent Femto Pulse Automated Pulsed Field CE Instrument (Agilent Technologies) using the gDNA 55 kb BAC analysis kit.

The sample was sequenced on a Revio instrument (Pacific Biosciences). The prepared library was normalised to 2 nM, and 15 μL was used for making complexes. Primers were annealed and polymerases bound to generate circularised complexes, following the manufacturer’s instructions. Complexes were purified using 1.2X SMRTbell beads, then diluted to the Revio loading concentration (200–300 pM) and spiked with a Revio sequencing internal control. The sample was sequenced on a Revio 25M SMRT cell. The SMRT Link software (Pacific Biosciences), a web-based workflow manager, was used to configure and monitor the run and to carry out primary and secondary data analysis.

***Hi-C***

For Hi-C library preparation, the biotinylated DNA constructs were fragmented using a Covaris E220 sonicator and size-selected to 400–600 bp using SPRISelect beads. DNA was then enriched using Arima-HiC v2 Enrichment beads. The NEBNext Ultra II DNA Library Prep Kit (New England Biolabs) was used for end repair, A-tailing, and adapter ligation, following a modified protocol in which library preparation is carried out while the DNA remains bound to the enrichment beads. PCR amplification was performed using KAPA HiFi HotStart mix and custom dual-indexed adapters (Integrated DNA Technologies) in a 96-well plate format. Depending on sample concentration and biotinylation percentage determined at the crosslinking stage, samples were amplified for 10–16 PCR cycles. Post-PCR clean-up was carried out using SPRISelect beads. The libraries were quantified using the Accuclear Ultra High Sensitivity dsDNA Standards Assay kit (Biotium) and normalised to 10 ng/μL before sequencing. Hi-C sequencing was performed on the Illumina NovaSeq X instrument using 150 bp paired-end reads.

### Genome assembly, curation and evaluation

***Assembly***

Prior to assembly of the PacBio HiFi reads, a database of *k*-mer counts ( *k* = 31) was generated from the filtered reads using FastK. GenomeScope2 ( Ranallo-Benavidez *et al.*, 2020) was used to analyse the *k*-mer frequency distributions, providing estimates of genome size, heterozygosity, and repeat content.

The HiFi reads were assembled using Hifiasm in Hi-C phasing mode ( Cheng *et al.*, 2021; Cheng *et al.*, 2022), resulting in a pair of haplotype-resolved assemblies. The Hi-C reads ( Rao *et al.*, 2014) were mapped to the primary contigs using bwa-mem2 ( Vasimuddin *et al.*, 2019). The contigs were further scaffolded using the provided Hi-C data in YaHS ( Zhou *et al.*, 2023) using the --break option for handling potential misassemblies. The scaffolded assemblies were evaluated using Gfastats ( Formenti *et al.*, 2022), BUSCO ( Manni *et al.*, 2021) and MERQURY.FK ( Rhie *et al.*, 2020).

The mitochondrial genome was assembled using MitoHiFi ( Uliano-Silva *et al.*, 2023), which runs MitoFinder ( Allio *et al.*, 2020) and uses these annotations to select the final mitochondrial contig and to ensure the general quality of the sequence.

***Assembly curation***

The assembly was decontaminated using the Assembly Screen for Cobionts and Contaminants (ASCC) pipeline. Flat files and maps used in curation were generated via the TreeVal pipeline ( Pointon *et al.*, 2023). Manual curation was conducted primarily in PretextView ( Harry, 2022) and HiGlass ( Kerpedjiev *et al.*, 2018), with additional insights provided by JBrowse2 ( Diesh *et al.*, 2023). Scaffolds were visually inspected and corrected as described by Howe *et al*. (2021). Any identified contamination, missed joins, and mis-joins were amended, and duplicate sequences were tagged and removed. Sex chromosomes were identified by synteny analysis. The curation process is documented at https://gitlab.com/wtsi-grit/rapid-curation. PretextSnapshot was used to generate a Hi-C contact map of the final assembly.

***Assembly quality assessment***

The Merqury.FK tool ( Rhie *et al.*, 2020), run in a Singularity container ( Kurtzer *et al.*, 2017), was used to evaluate *k*-mer completeness and assembly quality for both haplotypes using the *k*-mer databases ( *k* = 31) computed prior to genome assembly. The analysis outputs included assembly QV scores and completeness statistics.

The genome was analysed using the BlobToolKit pipeline, a Nextflow ( Di Tommaso *et al.*, 2017) implementation of the earlier Snakemake BlobToolKit pipeline ( Challis *et al.*, 2020). The pipeline aligns PacBio reads using minimap2 ( Li, 2018) and SAMtools ( Danecek *et al.*, 2021) to generate coverage tracks. Simultaneously, it queries the GoaT database ( Challis *et al.*, 2023) to identify relevant BUSCO lineages and runs BUSCO ( Manni *et al.*, 2021). For the three domain-level BUSCO lineages, BUSCO genes are aligned to the UniProt Reference Proteomes database ( Bateman *et al.*, 2023) using DIAMOND blastp ( Buchfink *et al.*, 2021). The genome is divided into chunks based on the density of BUSCO genes from the closest taxonomic lineage, and each chunk is aligned to the UniProt Reference Proteomes database with DIAMOND blastx. Sequences without hits are chunked using seqtk and aligned to the NT database with blastn ( Altschul *et al.*, 1990). The BlobToolKit suite consolidates all outputs into a blobdir for visualisation.

The BlobToolKit pipeline was developed using nf-core tooling ( Ewels *et al.*, 2020) and MultiQC ( Ewels *et al.*, 2016), with package management via Conda and Bioconda ( Grüning *et al.*, 2018), and containerisation through Docker ( Merkel, 2014) and Singularity ( Kurtzer *et al.*, 2017).

Table 4 contains a list of relevant software tool versions and sources.

**Table 4.** Software tools: versions and sources.

Software tool	Version	Source
BLAST	2.14.0	ftp://ftp.ncbi.nlm.nih.gov/blast/executables/blast+/
BlobToolKit	4.3.9	https://github.com/blobtoolkit/blobtoolkit
BUSCO	5.5.0	https://gitlab.com/ezlab/busco
bwa-mem2	2.2.1	https://github.com/bwa-mem2/bwa-mem2
DIAMOND	2.1.8	https://github.com/bbuchfink/diamond
fasta_windows	0.2.4	https://github.com/tolkit/fasta_windows
FastK	666652151335353eef2fcd58880bcef5bc2928e1	https://github.com/thegenemyers/FASTK
GenomeScope2.0	2.0.1	https://github.com/tbenavi1/genomescope2.0
Gfastats	1.3.6	https://github.com/vgl-hub/gfastats
GoaT CLI	0.2.5	https://github.com/genomehubs/goat-cli
Hifiasm	0.19.8-r603	https://github.com/chhylp123/hifiasm
HiGlass	44086069ee7d4d3f6f3f0012569789ec138f42b84aa44357826c0b6753eb28de	https://github.com/higlass/higlass
MerquryFK	d00d98157618f4e8d1a9190026b19b471055b22e	https://github.com/thegenemyers/MERQURY.FK
Minimap2	2.24-r1122	https://github.com/lh3/minimap2
MitoHiFi	3	https://github.com/marcelauliano/MitoHiFi
MultiQC	1.14, 1.17, and 1.18	https://github.com/MultiQC/MultiQC
Nextflow	23.10.0	https://github.com/nextflow-io/nextflow
PretextView	0.2.5	https://github.com/sanger-tol/PretextView
samtools	1.19.2	https://github.com/samtools/samtools
sanger-tol/ascc	0.1.0	https://github.com/sanger-tol/ascc
sanger-tol/blobtoolkit	0.6.0	https://github.com/sanger-tol/blobtoolkit
Seqtk	1.3	https://github.com/lh3/seqtk
Singularity	3.9.0	https://github.com/sylabs/singularity
TreeVal	1.2.0	https://github.com/sanger-tol/treeval
YaHS	1.2a.2	https://github.com/c-zhou/yahs

### Wellcome Sanger Institute – Legal and Governance

The materials that have contributed to this genome note have been supplied by a Darwin Tree of Life Partner. The submission of materials by a Darwin Tree of Life Partner is subject to the **‘Darwin Tree of Life Project Sampling Code of Practice’**, which can be found in full on the Darwin Tree of Life website here. By agreeing with and signing up to the Sampling Code of Practice, the Darwin Tree of Life Partner agrees they will meet the legal and ethical requirements and standards set out within this document in respect of all samples acquired for, and supplied to, the Darwin Tree of Life Project.

Further, the Wellcome Sanger Institute employs a process whereby due diligence is carried out proportionate to the nature of the materials themselves, and the circumstances under which they have been/are to be collected and provided for use. The purpose of this is to address and mitigate any potential legal and/or ethical implications of receipt and use of the materials as part of the research project, and to ensure that in doing so we align with best practice wherever possible. The overarching areas of consideration are:

•    Ethical review of provenance and sourcing of the material

•    Legality of collection, transfer and use (national and international)

Each transfer of samples is further undertaken according to a Research Collaboration Agreement or Material Transfer Agreement entered into by the Darwin Tree of Life Partner, Genome Research Limited (operating as the Wellcome Sanger Institute), and in some circumstances other Darwin Tree of Life collaborators.

## Data Availability

European Nucleotide Archive: Larinioides cornutus. Accession number PRJEB82167; https://identifiers.org/ena.embl/PRJEB82167. The genome sequence is released openly for reuse. The *Larinioides cornutus* genome sequencing initiative is part of the Darwin Tree of Life Project (PRJEB40665). All raw sequence data and the assembly have been deposited in INSDC databases. The genome will be annotated using available RNA-Seq data and presented through the Ensembl pipeline at the European Bioinformatics Institute. Raw data and assembly accession identifiers are reported in Table 1 and Table 2.

## Author information

Members of the University of Oxford and Wytham Woods Genome Acquisition Lab are listed here: https://doi.org/10.5281/zenodo.12157525.

Members of the Darwin Tree of Life Barcoding collective are listed here: https://doi.org/10.5281/zenodo.12158331.

Members of the Wellcome Sanger Institute Tree of Life Management, Samples and Laboratory team are listed here: https://doi.org/10.5281/zenodo.12162482.

Members of Wellcome Sanger Institute Scientific Operations: Sequencing Operations are listed here: https://doi.org/10.5281/zenodo.12165051.

Members of the Wellcome Sanger Institute Tree of Life Core Informatics team are listed here: https://doi.org/10.5281/zenodo.12160324.

Members of the Tree of Life Core Informatics collective are listed here: https://doi.org/10.5281/zenodo.12205391.

Members of the Darwin Tree of Life Consortium are listed here: https://doi.org/10.5281/zenodo.4783558.
