# Supplementary material for: The genome sequence of the furrow orbweaver, Larinioides cornutus (Clerck, 1757)
Source: Wellcome Open Res. 2025 May 23;10:267. [Version 1] doi: 10.12688/wellcomeopenres.24288.1 (PMC12586987; doi:10.12688/wellcomeopenres.24288.1)
Supplement: Peer review history

# Reviewer report: Reviewer response for version 1

**DOI:** 10.21956/wellcomeopenres.26786.r126103

**Published:** 2025-11-05

**Authors:** Gabriela Pozo

**Affiliations:** 1 Universidad San Francisco de Quito, Quito, Ecuador

This manuscript presents a haplotype-resolved genome assembly for a spider species, generated using long-read sequencing and phased with Hi-C data. The authors report a genome size of approximately 2,258.74 Mb and provide assemblies for two haplotypes. The study is well-structured, with clearly described methods, informative results, and public accessibility of the genomic data. This genome represents an important contribution to arachnid genomics and provides a valuable resource for future studies of spider biology, evolution, and comparative genomics.

 I have two suggestions for the authors:

 1. You mention that this study will contribute to broader studies of the biology and phylogeny of the order Araneae. I suggest briefly stating here what the current state of sequenced genomes in this order is, if there is representatitvity or not.

 2. In table 2, I suggest stating how many BUSCO groups the database you used has (arachnida_odb10), like you do for the snailplot.

Are sufficient details of methods and materials provided to allow replication by others?

Yes

Is the rationale for creating the dataset(s) clearly described?

Partly

Are the datasets clearly presented in a useable and accessible format?

Yes

Are the protocols appropriate and is the work technically sound?

Yes

Reviewer Expertise:

Population genetics, genomics

I confirm that I have read this submission and believe that I have an appropriate level of expertise to confirm that it is of an acceptable scientific standard.

# Reviewer report: Reviewer response for version 1

**DOI:** 10.21956/wellcomeopenres.26786.r126100

**Published:** 2025-11-04

**Authors:** Jesús A Ballesteros

**Affiliations:** 1 Kean University, Union, New Jersey, USA

This manuscript presents the chromosome genome assembly of the furrow spider "Lariniodes cornutus". This orbweaving spider, common across the holartic region, represents a valuable genomic resource for the broad scientific community. The dataset presented in this contribution can be readily useful across various biological disciplines: population genetics, phylogenetics, silk, venom, etc. The detail and clarity in the method descriptions are also very valuable for future projects targeting non model organism. In consequence I believe the manuscript is indexable in its current format.

Are sufficient details of methods and materials provided to allow replication by others?

Yes

Is the rationale for creating the dataset(s) clearly described?

Yes

Are the datasets clearly presented in a useable and accessible format?

Yes

Are the protocols appropriate and is the work technically sound?

Yes

Reviewer Expertise:

arachnids, phylogenetics, bioinformatics

I confirm that I have read this submission and believe that I have an appropriate level of expertise to confirm that it is of an acceptable scientific standard.

# Reviewer report: Reviewer response for version 1

**DOI:** 10.21956/wellcomeopenres.26786.r124283

**Published:** 2025-06-23

**Authors:** Peng-Yu Jin

**Affiliations:** 1 Institute of Zoology, Chinese Academy of Sciences, Beijing, Beijing, China

The manuscript presents a high-quality chromosomal-level genome assembly of *Larinioides cornutus*, an ecologically widespread orb-weaving spider. They generated a robust dataset using PacBio HiFi and Hi-C, achieving a highly contiguous assembly. Quality assessments indicate excellent completeness and accuracy. The study follows current community standards for genome assembly, including EBP reference standard, and provides sufficient methodological transparency to ensure reproducibility. Overall, this is a strong contribution and merits publication.

Are sufficient details of methods and materials provided to allow replication by others?

Yes

Is the rationale for creating the dataset(s) clearly described?

Yes

Are the datasets clearly presented in a useable and accessible format?

Yes

Are the protocols appropriate and is the work technically sound?

Yes

Reviewer Expertise:

Spider evolution; Single-cell RNA-seq; Spider genomics

I confirm that I have read this submission and believe that I have an appropriate level of expertise to confirm that it is of an acceptable scientific standard.
